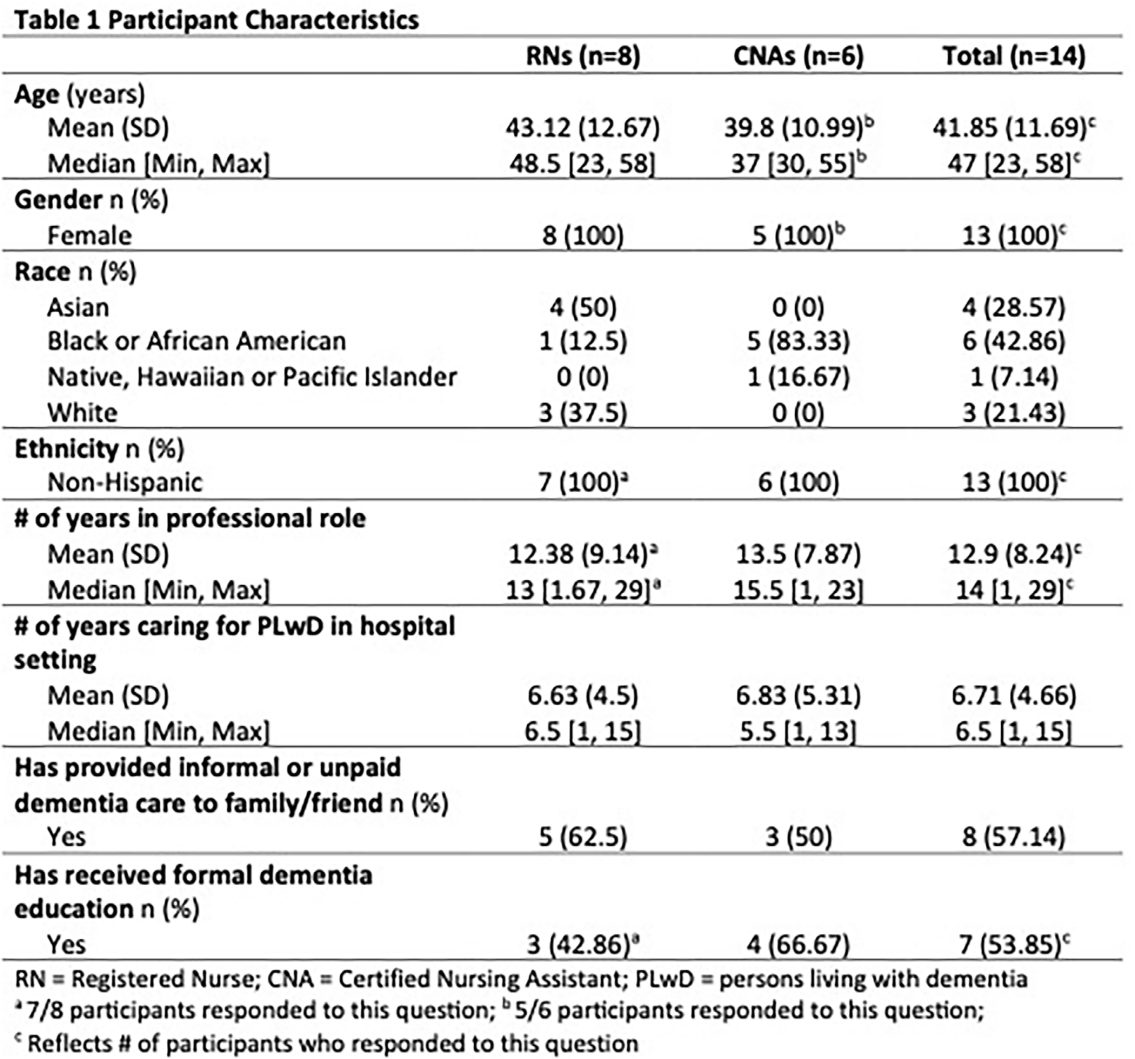# Use of the hybrid model for developing the concept of dementia friendly in the context of hospitalization

**DOI:** 10.1002/alz.093146

**Published:** 2025-01-09

**Authors:** Ellen Munsterman, Pamela Z Cacchione

**Affiliations:** ^1^ University of Pennsylvania, Philadelphia, PA USA

## Abstract

**Background:**

Persons living with dementia (PLwD) are hospitalized at greater rates than adults without dementia and experience more adverse outcomes. The use of dementia friendly practices is increasingly common, but definitions for the concept vary. This study describes the fieldwork phase of a hybrid approach to concept development. This phase followed an analysis of the literature that resulted in a working definition for the concept of dementia friendly in the context of hospitalization. This definition incorporates the major recurring attributes found in the literature, including dementia‐related knowledge and education, environmental modifications, person‐centered care, the role of nursing care, and the inclusion of family caregivers. The aim of this study was to refine and confirm the working definition for the concept of dementia friendly in the context of hospitalization, informed by the perceptions of Registered Nurses (RNs) and Certified Nursing Assistants (CNAs).

**Methods:**

Semi‐structured interviews were conducted with RNs and CNAs employed on an Acute Care for Elders unit. Interviews were audio‐recorded and professionally transcribed. Directed content analysis was used to analyze the interview data. Transcriptions were coded independently by the two authors and reconciled until agreement was reached.

**Results:**

Participant demographics are reported in Table 1. Participants were largely in agreement with the definition as written but contributed important nuance to several of the attributes. Regarding staff knowledge and education, participants highlighted the role of hands‐on experience, as well as the delivery and format: synchronous vs. asynchronous. They stressed other characteristics required to coincide with knowledge, including care, compassion, and patience. When discussing the inclusion of family caregivers, participants addressed challenges faced when visiting family members lacked dementia‐specific knowledge.

**Conclusion:**

The hybrid approach to concept development is useful for refining complex concepts pertinent to the field of nursing, enabling measurement and further research. In this study, combining qualitative fieldwork with a review of the literature resulted in the refinement of a working definition of the concept of dementia friendly in the context of hospitalization. Dementia friendly practices are necessary to address the unique needs of PLwD in the hospital. A clear definition of the concept will facilitate widespread implementation.